# Recurrent, bilateral endogenous *Candida* endophthalmitis with multiple focal chorioretinal lesions: management with pars plana vitrectomy and focal endolaser

**DOI:** 10.1186/s12348-022-00301-6

**Published:** 2022-07-11

**Authors:** Prashanth G. Iyer, Jason Fan, J. Daniel Diaz, Jeremy Liu, Thomas Lazzarini, Kenneth C. Fan, Darleen Miller, Harry W. Flynn

**Affiliations:** Department of Ophthalmology, Bascom Palmer Eye Institute, University of Miami Miller School of Medicine, 900 NW 17th St, Miami, FL 33136 USA

**Keywords:** Candida, Endophthalmitis, Endogenous endophthalmitis, Fungal endophthalmitis, Vitrectomy, Endolasser

## Abstract

**Purpose:**

Bilateral endogenous *Candida* endophthalmitis (ECE) treatment usually involves administering systemic and intravitreal antifungal medications. In advanced cases with vitreous seeding, pars plana vitrectomy (PPV) is considered. The use of focal endolaser treatment to chorioretinal lesions has not been reported. We present a case of bilateral recurrent ECE treated with PPV and endolaser to elevated focal lesions.

**Case:**

A 45-year-old diabetic male presented with decreased visual acuity in both eyes (20/50 right eye, 20/150 left eye) and was found to have bilateral ECE with moderate vitritis and chorioretinal lesions. The initial treatment consisted of multiple intravitreal voriconazole injections to both eyes as well as systemic antifungal therapy. Resolution of ECE occurred after three months, but one year later despite therapy recurred bilaterally. Patient underwent PPV with endolaser to the elevated chorioretinal lesions in both eyes. One year later, his vision improved to 20/40 in both eyes, focal lesions were flat and resolved along with the ECE.

**Conclusion:**

Advanced or recurrent ECE that is refractive to intravitreal antifungal therapy may be treated with PPV. Endolaser therapy to the chorioretinal lesions is an additional local option that can resolve the activity of ECE.

## Introduction

Endogenous endophthalmitis accounts for between 2 and 15% of all causes of endophthalmitis, of which approximately 50% are fungal [1]. Endogenous fungal endophthalmitis (EFE) is often associated with poor visual outcomes and most commonly affects immunocompromised individuals [1, 2]. *Candida* species, which contribute to the human flora found in mucosal surfaces of the respiratory, gastrointestinal and female genital tracts, are the most common causes of EFE [3, 4]. *Candida albicans,* the most common cause of Candidemia, can result in chorioretinitis and endophthalmitis via hematogenous seeding [3]. In fact, bilateral endogenous endophthalmitis secondary to yeasts was identified in 30% of positive cultures in EFE cases in one series [4]. Treatment of endogenous *Candida* endophthalmitis (ECE) is primarily through systemic administration of antifungal agents. Intravitreal antifungal administration and/or vitrectomy are considered when there is advanced vitritis or macular threatening lesions [5]. We present a unique management method of a patient with advanced bilateral ECE and multiple intraretinal lesions using PPV and focal endolaser as well as systemic and intravitreal antifungal medications.

## Case Report

45-year-old Hispanic, diabetic male presented with decreased vision, pain, and conjunctival injection in both eyes. He denied any intravenous drug use, recent travel, trauma, sexually transmitted diseases, malignancy or immunocompromised state. His most striking risk factors included type 1 diabetes mellitus and bilateral shoulder abscesses surgically removed four months prior to onset of visual symptoms. The visual acuity on presentation was 20/50 in the right eye, and 20/150 in the left eye. Examination demonstrated bilateral EFE, and blood cultures confirmed growth for *Candida albicans.* Initial management included intravitreal vancomycin (1 mg/0.1 ml), ceftazidime (2.25 mg/0.1 ml) and voriconazole (100 μg/0.1 ml) in both eyes. The patient was started on oral fluconazole 600 mg daily for four weeks and received four additional intravitreal voriconazole injections to both eyes. Three months after his last injection, there was improvement of the vitritis and chorioretinitis with visual acuity returning to baseline of 20/25 in both eyes.

**Fig. 1:**
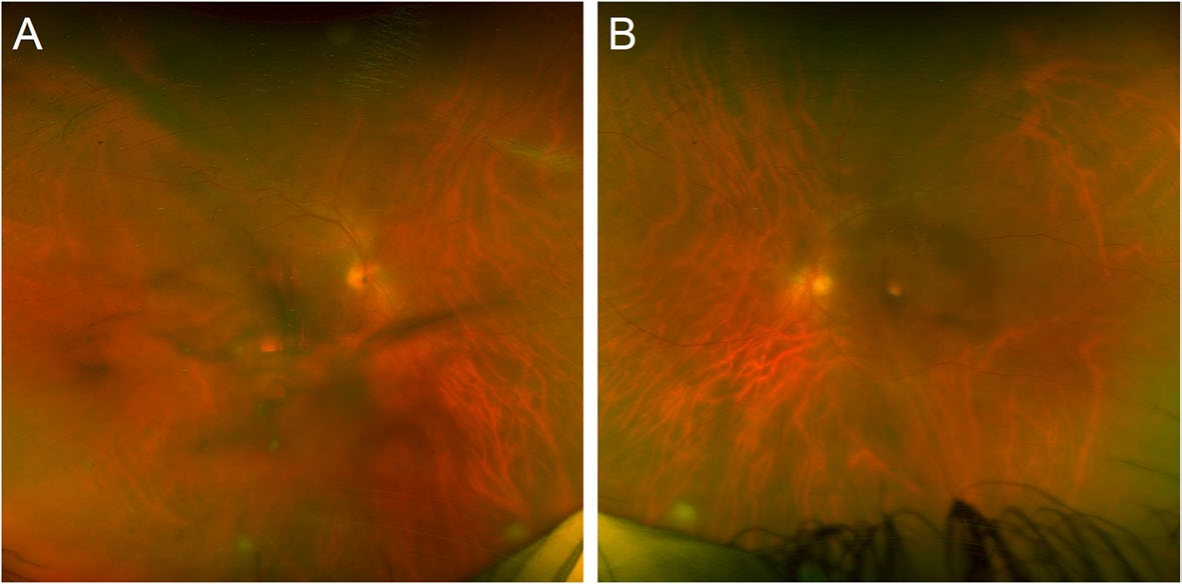
(**A** and **B**) Bilateral recurrent endogenous *Candida* endophthalmitis (ECE) is seen with moderate vitritis and chorioretinal lesions one year after failing systemic and intravitreal antifungal therapy bilaterally

**Fig. 2:**
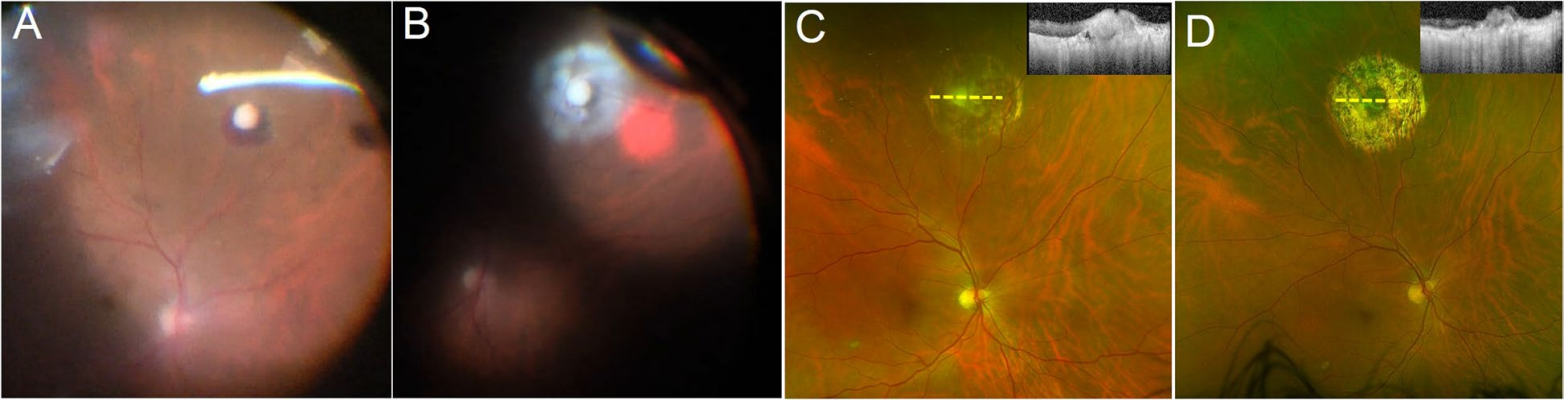
(**A** and **B**): Recurrent endogenous Candida endophthalmitis (ECE) of the right eye is demonstrated. **A** Moderate vitritis and a chorioretinal lesion is present in the right eye seen during pars plana vitrectomy. **B** The chorioretinal lesion in the right eye is lasered directly, along with rows of laser surrounding the lesion. **C** One months after pars plana vitrectomy, focal laser and intravitreal voriconazole to the right eye, the lesion has become scarred; optical coherence tomography (OCT) through the lesions (yellow line) demonstrates scarring of the chorioretinal lesion. **D** One year after surgery, the endolaser scarring has become pigmented; OCT through the lesion (yellow line) demonstrates atrophy and scarring of the chorioretinal lesion

One year later, the patient returned with pain and blurry vision in both eyes. Visual acuity was 20/40 in both eyes. The examination revealed recurrent moderate vitritis and chorioretinal lesions in both eyes (Fig. 1). Oral voriconazole 600 mg daily was restarted by the infectious disease consultant. He received two intravitreal injections of voriconazole in both eyes but had not improvement. At that time, his visual acuity dropped to 20/100 in the right eye and 20/200 in the left eye. Given the lack of initial improvement of the vitreous infiltration and worsening vision, the patient underwent 25-gauge core vitrectomy and vitreous base shaving. The right eye had surgery first and one week later, the left eye underwent vitrectomy. The vitreous specimens were sent for culture in both eyes, which confirmed *Candida albicans*. During surgery, focal endolaser was applied to each chorioretinal lesion in both eyes (Fig. 2A and B). Intravitreal voriconazole was administered at the end of surgery to each eye. The patient resumed oral fluconazole under the guidance of the infectious disease physicians. At one year after vitrectomy surgery, visual acuity improved to 20/25 in both eyes, with resolution of vitritis. The lesions that had laser application demonstrated atrophy and scarring (Fig. 2D). OCT performed through one of the laser-treated lesions in the right eye showed slight elevation and fibrosis.

## Discussion

ECE occurs in 2.5% of patients with disseminated *Candidemia*, though this prevalence rate has decreased more recently likely due to earlier detection and treatment of *Candida* when detected in cultures [6]. Risk factors for ECE include recent hospitalization, recent surgery, gastrointestinal procedure, diabetes mellitus, immunosuppression, intravenous drug use, indwelling catheters, glucocorticoid therapy, and history of transplant [4, 7, 8]. In addition, neonates, patients with malignancy, and burn patients are particularly prone to fungal dissemination [7].

Approximately 14–33% of patients with endogenous endophthalmitis have positive fungal blood cultures [9]. In patients with *Candidemia*, the incidence of chorioretinal lesions has been reported to be 11%, while the incidence of endophthalmitis with vitreous involvement is 1.6% [9]. Around 80% of patients with ECE have multifocal lesions [9]. Complications of these chorioretinal lesions in ECE with worsening intraocular inflammation include tractional and rhegmatogenous retinal detachments as well as cyclitic membrane formation, a combination of which can results in pthisis bulbi [10]. The patient in the current report had positive blood cultures for *Candida albicans* and bilateral posterior segment disease with multifocal chorioretinal lesions.

Recurrent ECE has been described in multiple case reports and series [11, 12]. However, there is no consensus on the management of ECE. Treatment generally involves both intravitreal anti-fungal agents as well as systemic antifungal therapy depending on blood cultures and systemic symptoms. Systemic agents such as intravenous voriconazole, fluconazole or amphotericin B may be adequate for the treatment of non-macula-threatening chorioretinitis, whereas endophthalmitis and macula-threatening chorioretinitis require both systemic therapy and intravitreal injections. Intravitreal options include voriconazole (50–100 μg/0.1 ml), amphotericin B (5–10 μg/0.1 ml), miconazole (25 μg/0.1 ml) and echinocandins. Intravitreal voriconazole is generally well tolerated, and can even be repeated if necessary [7]. Several reports have demonstrated the safety and efficacy of intravitreal voriconazole in EFE [13, 14].

Outcomes of pars plana vitrectomy (PPV) in ECE were first reported in 1976; Snip and Michel described the rapid clearing of intraocular infection after vitrectomy and intravitreal amphotericin B [10, 15]. Peyman also successfully treated a patient with ECE after trauma with PPV and amphotericin B [10, 16]. In one series of endogenous *Candida* endophthalmitis without systemic disseminated disease, patients were successfully treated with PPV and intravitreal amphotericin B without systemic therapy [10]. In a series of patients with EFE secondary to urinary infections after procedure, the majority of patients had an improvement in visual acuity after undergoing PPV with intravitreal amphotericin B and oral fluconazole [17]. In another study, patients were initially managed with systemic antifungals and intravitreal amphotericin B, however PPV was utilized in patients with worsening intraocular inflammation [18]. One large case study advocated for a conservative approach for EFE compared to endogenous bacterial endophthalmitis [19].

The use of argon green 532 nm endolaser to treat chorioretinal candida lesions has not been described in the literature to our knowledge. The rationale for using laser to treat fungal lesions has been described in other subspecialties in medicine. Onychomycosis has been treated with femtosecond laser successfully, inhibiting growth of fungus in all cases [20]. Fungal-related dental cavities due to candida have been successfully treated with argon laser for 12–16 minutes along with nystatin [21]. Laser irradiation has been shown to also inhibit in vitro growth of *Trichophyton rubrum*, a common fungus isolate found in skin, hair and nails [22]. Two cases of *Fusarium* keratitis treated with direct focal argon laser to affected areas, demonstrated complete resolution of the infiltrates and without adverse effects [23]. Also, photodynamic therapy has been shown to inhibit growth of multi-resistant organisms effectively in keratitis [24]. The above mentioned studies supports our rationale to directly laser the chorioretinal lesions in ECE, and then apply rows of laser around the lesion. This technique may help to prevent chorioretinitis-related complications such a rhegmatogenous and tractional retinal detachments, and well as irradiate persistent disease. Our patient with bilateral ECE did well with PPV, selective focal endolaser, and intravitreal voriconazole, with no recurrence of disease after one year of follow-up. More experience will be required to support this hypothesis of using focal laser to treat chorioretinal lesions in ECE.
